# Supplementary material for: Simultaneous Determination of 108 Pesticide Residues in Three Traditional Chinese Medicines Using a Modified QuEChERS Mixed Sample Preparation Method and HPLC-MS/MS
Source: Molecules. 2022 Nov 7;27(21):7636. doi: 10.3390/molecules27217636 (PMC9658470; doi:10.3390/molecules27217636)
Supplement: Supplementary file 1 [file molecules-27-07636-s001.zip › molecules-2006544-supplementary.pdf]

Supplemental materials

Simultaneous Determination of 108 Pesticide Residues in Three Traditional Chinese Medicines Using a Modified QuEChERS Mixed Sample Preparation Method and HPLC-MS/MS

Xuyan Fan [1,2], Tao Tang [2], Song Du [1,2], Ningning Sang [2], Hao Huang [2], Chenghui Zhang [1,3,*] and Xueping Zhao [2,*]

1 College of Food Science and Engineering, Hainan University, Haikou 570228, China
2 State Key Laboratory for Managing Biotic and Chemical Threats to the Quality and Safety of Agro-Products, Key Laboratory of Detection for Pesticide Residues and Control of Zhejiang, Institute of Agro-Product Safety and Nutrition, Zhejiang Academy of Agricultural Sciences, Hangzhou 310021, China
3 Key Laboratory of Tropical Fruits and Vegetables Quality and Safety for State Market Regulation, Haikou 570206, China
* Correspondence: zchlm@hainanu.edu.cn (C.Z.); zhaoxueping@tom.com (X.Z.); Tel.: +86-571-85273092 (X.Z.)

Table S1. Parameters of 108 pesticides determined by HPLC-MS/MS.

No.	Pesticides	Retention Time (min)	Parent ion (m/z)	Daughter ion 1 (m/z)	CE 1 (eV)	Daughter ion 2 (m/z)	CE 1 (eV)
1	Betaine	1.344	118.2	58.15	-30	59.15	-19
2	Acephate	3.502	184.2	143.0	-8	95.0	-23
3	Omethoate	3.894	214.1	183.0	-10	155.0	-14
4	3-Indoleacetic acid	5.723	176.1	130.2	-15	103.2	-32
5	Aldicarb sulfoxide	4.209	207.1	89.0	-14	132.0	-8
6	Dinotefuran	4.189	203.1	129.1	-12	113.1	-22
7	Propamocarb	4.43	189.2	144.05	-20	102.5	-12
8	Aldicarb sulfone	4.588	240.1	223.0	-21	86.0	-8
9	Methomyl	5.169	163.05	88.0	-8	106.05	-10
10	Deisopropylatrazine	6.047	173.7	68.1	-26	104.15	-23
11	Thiamethoxam	5.403	292.0	211.1	-11	181.1	-23
12	Gibberellic acid	5.623	345.15	143.1	30	221.15	24
13	Abscisic Acid	7.311	263.1	153.2	10	204.1	17
14	Pyrazosulfuron-Ethyl	8.907	415.1	182.05	-18	139.05	-42
15	Cycloxaprid	6.014	323.1	126.05	-34	277.1	-14
16	Clothianidin	6.377	250.0	131.85	-10	169.0	-14
17	Imidacloprid	6.278	256.05	175.1	-17	209.05	-14
18	Trichlorfon	6.847	256.9	108.95	-17	220.85	-10
19	3-Hydroxycarbofuran	6.797	238.1	163.1	-14	181.2	-10
20	6-Kinetin	7.005	216.0	81.1	-22	148.1	-13
21	Sodium 5-Nitroguaiacolate	7.291	167.9	153.1	16	123.2	21
22	Sodium 2-Nitrophenolate	7.344	138.15	108.1	19	92.1	21
23	Sodium 4-Nitrophenolate	7.338	138.15	108.1	19	92.0	24
24	2,4-D	8.054	219.0	161.1	13	125.0	25
25	Dimethoate	6.793	230.0	198.95	-9	125.0	-22
26	Acetamiprid	6.796	223.1	126.05	-22	56.1	-15
27	Carbendazim	7.323	192.05	160.05	-17	132.05	-30
28	Thiacloprid	7.273	253.0	126.05	-20	99.0	-43
29	Trinexapac-Ethyl	8.787	253.1	69.1	-23	207.1	-12
30	Tricyclazole	7.578	190.0	136.0	-26	163.0	-21
31	Indolbutyric Acid	8.074	204.2	186.25	-13	130.3	-14
32	6-Benzylaminopurine	8.343	226.0	91.1	-30	65.2	-52
33	Thidiazuron	8.844	221.1	102.1	-16	128.0	-15
34	Dichlorvos	8.863	221.0	109.1	-16	79.1	-27
35	Carbofuran	8.966	222.1	123.1	-11	165.1	-21
36	Carbaryl	9.385	202.05	145.05	-9	127.05	-27
37	Pirimicarb	10.015	239.15	72.05	-25	182.15	-19
38	Forchlorfenuron	10.501	248.1	129.1	-17	93.1	-22
39	RH-5849	9.812	295.1	121.1	18	77.25	32

40	Isopropyl	10.319	194.1	95.0	-14	137.1	-17
41	Atrazine	10.319	216.1	174.05	-17	96.05	-25
42	Imidan	11.065	318.0	160.0	-13	77.1	-54
43	Chlorantraniliprole	10.942	484.0	452.9	-19	285.85	-16
44	Clomazone	11.205	240.1	125.0	-19	89.1	-50
45	Azoxystrobin	11.336	404.1	372.05	-14	329.0	-31
46	Methyl Jasmonate	11.573	225.2	133.2	-12	151.2	-14
47	Pyrimethanil	11.735	200.1	107.0	-25	168.1	-29
48	Fenamidone	11.756	312.1	92.05	-15	236.1	-24
49	Boscalid	11.893	343.0	307.1	-18	271.1	-30
50	Dimethomorph	12.177	388.1	301.0	-20	165.05	-34
51	Mandipropamid	11.918	412.1	328.05	-12	124.95	-30
52	Isoprothiolane	12.107	291.1	231.1	-11	189.1	-21
53	Fluopicolide	12.188	382.9	172.95	-22	145.0	-47
54	Paclobutrazol	12.068	294.1	70.5	-21	125.05	-40
55	Uniconazole	12.712	292.1	70.1	-24	125.0	-28
56	Malathion	12.107	331.0	99.0	-12	127.05	-23
57	Orysastrobin	12.172	392.10	205.1	-18	116.1	-28
58	Benthiavalicarb-Isopropyl	12.189	382.1	180.1	-31	116.1	-20
59	Methoxyfenozide	12.184	369.2	149.1	-16	313.1	-8
60	Triadimefon	12.318	294.1	69.15	-22	197.05	-15
61	Tiadinil	12.53	265.8	71.0	16	238.0	12
62	Cyproconazole	12.717	292.1	70.05	-20	125.05	-30
63	Triazophos	12.508	314.05	162.15	-19	119.15	-35
64	Fenhexamid	12.803	301.9	97.1	-52	55.1	-26
65	Chromafenozide	12.625	395.3	175.1	-16	339.15	-7
66	Tetrachlorantraniliprole	12.801	539.5	319.9	-17	508.3	-15
67	Spirotetramat	12.684	374.15	302.1	-16	330.2	-15
68	Epoxiconazole	12.962	330.1	121.2	-21	141.1	-18
69	Furan Tebufenozide	12.644	395.0	175.0	-35	339.0	-20
70	Diclocymet	13.014	311.0	96.0	17	80.1	34
71	Alachlor	13.081	270.1	238.05	-10	162.15	-19
72	Tetraconazole	12.978	372.0	129.05	-31	70.2	-24
73	Cyazofamid	13.041	325.0	108.05	-12	261.1	-10
74	Fenoxanil	13.471	329.1	302.1	-12	86.1	-22
75	Picoxystrobin	13.427	368.2	144.95	-21	205.15	-9
76	Flusilazole	13.394	316.1	165.1	-18	247.1	-29
77	Kresoxim-Methyl	13.665	314.1	225.15	-16	235.15	-16
78	Fipronil	13.449	435.0	330.0	16	250.0	28
79	Diazinon	14.036	305.0	169.1	-19	153.1	-20
80	Brassinolide	14.143	481.3	445.4	-14	315.3	-16
81	Tebuconazole	13.881	308.1	70.1	-22	125.0	-38
82	Pentrimazole	13.834	284.1	70.0	-27	159.0	-17

83	Pythiamin	13.818	360.0	276.0	-15	177.0	-34
84	Propiconazole	14.052	342.05	159.1	-30	205.1	-18
85	Pyraoxystrobin	14.207	413.1	205.1	-18	145.0	-10
86	Pyraclostrobin	14.191	387.8	162.9	-13	193.7	-23
87	Phoxim	14.227	299.0	77.1	-26	129.1	-10
88	Cyflufenamid	14.361	413.2	295.05	-16	203.0	-40
89	Diniconazole	14.733	326.1	70.0	-25	159.0	-30
90	Ametoctradin	14.908	276.2	149.0	-35	176.1	-35
91	Indoxacarb	14.864	528.1	249.1	-17	292.95	-15
92	Pretilachlor	15.148	312.2	252.15	-16	176.15	-28
93	Difenoconazole	14.794	406.1	251.0	-25	337.05	-17
94	Trifloxystrobin	14.943	409.1	186.05	-18	145.0	-44
95	Profenofos	15.442	372.9	302.8	-19	345.0	-12
96	Enestroburin	15.551	100.1	137.0	-25	178.0	-15
97	Coumoxystrobin	15.756	437.1	145.1	-35	205.1	-10
98	Buprofezin	15.805	306.1	201.1	-11	116.1	-16
99	Emamectin Benzoate	16.566	886.6	158.15	-40	126.3	-40
100	Fenpropathrin	16.219	350.3	97.2	-18	125.2	-31
101	Chlorpyrifos	16.213	351.9	199.9	-18	96.95	-33
102	Hexythiazox	16.215	353.1	228.0	-15	168.05	-25
103	Propargite	16.524	368.2	231.2	-11	175.15	-17
104	Spirodiclofen	16.796	411.1	71.2	-16	313.05	-11
105	Pyridaben	17.28	365.1	147.1	-12	309.05	-25
106	Carbosulfan	18.042	381.2	118.1	-19	160.1	-14
107	Etofenprox	18.441	394.0	177.1	-17	106.95	-40
108	Bifenthrin	18.754	440.3	181.1	-21	166.2	-43

Table S2. Sets of experiments of different dosages of sorbents.

Group	Sorbents	Outer diameter	Length
1	10 mg MWCNT(short) + 150 mg $MgSO_4$	OD：20 - 30 nm	L：0.5 - 2 μm
2	10 mg MWCNT(short) + 50 mg $MgSO_4$	OD：10 - 20 nm	L：0.5 - 2 μm
3	10 mg MWCNT(long) + 150 mg $MgSO_4$	OD：10 - 20 nm	L：10 - 30 μm
4	10 mg g-MWCNTs + 150 mg $MgSO_4$	OD：10 - 20 nm	L：5 - 30 μm
5	10 mg g-MWCNTs + 150 mg $MgSO_4$	OD：30 - 50 nm	L：10 - 20 μm
6	10 mg MWCNTs-COOH + 150 mg $MgSO_4$	OD：30 - 50 nm	L：10 - 20 μm
7	10 mg MWCNTs-NH_2 + 150 mg $MgSO_4$	OD：30 - 50 nm	L：10 - 20 μm
8	10 mg MWCNTs-OH + 150 mg $MgSO_4$	OD：8 - 15 nm	L：~ 50 μm
9	10 mg C18 + 150 mg $MgSO_4$		
10	50 mg PSA + 150 mg $MgSO_4$		
11	50 mg PSA + 50 mg C18+150 mg $MgSO_4$		
12	10 mg ZrO_2 + 150 mg $MgSO_4$		

Table S3. Calibration curves, correlation coefficients (R^2), limit of detection (LOD), limit of quantification (LOQ), and matrix effects for each target pesticide in *Fritillaria thunbergii* Miq.

No.	Pesticide	Calibration curves	R^2	LOD (μg/kg)	LOQ (μg/kg)	ME (%)
1	Betaine	y=14468x+24362	0.9997	0.33	1.11	198.65
2	Acephate	y=34023x+7615.2	0.9998	0.40	1.32	200.08
3	Omethoate	y=47297x-16876	0.9997	0.73	2.42	164.55
4	3-Indoleacetic acid	y=13594x-10839	0.9995	0.11	0.37	37.13
5	Aldicarb sulfoxide	y=12203x-1129.3	0.9997	0.38	1.25	12.29
6	Dinotefuran	y=16697x+32608	0.9994	0.49	1.65	9.8
7	Propamocarb	y=16551x-13730	0.9995	0.10	1.33	-15.72
8	Aldicarb sulfone	y=41309x+33389	0.9999	0.47	1.57	3.12
9	Methomyl	y=13935x+1770.8	0.9999	0.17	0.56	-100
10	Deisopropylatrazine	y=6854.6x+19245	0.9989	0.53	1.76	49.06
11	Thiamethoxam	y=42520x+51991	0.9995	0.26	0.88	11.11
12	Gibberellic acid	y=974.34x-850.04	0.9992	1.98	6.61	-22.15
13	Abscisic Acid	y=2233.6x-2163.5	0.9996	0.15	0.51	-13.96
14	Pyrazosulfuron-Ethyl	y=17501x-13369	0.9994	0.16	0.52	34.99
15	Cycloxaprid	y=23808x+12288	1	0.22	0.73	-7.75
16	Clothianidin	y=11914x+3826.4	0.9994	0.38	1.26	40.25
17	Imidacloprid	y=22255x-3224.9	0.9992	0.93	3.09	77.95
18	Trichlorfon	y=14281x+28320	0.9992	0.60	1.99	92.80
19	3-Hydroxycarbofuran	y=9956x+18768	0.9992	0.84	2.82	151.36
20	6-Kinetin	y=92056x+38924	0.9995	1.29	4.29	130.56

21	Sodium 5-Nitroguaiacolate	y=1545.3x+1351.7	0.9997	1.10	3.65	-3.74
22	Sodium 2-Nitrophenolate	y=4453.5x+10613	0.9999	0.42	1.39	17.13
23	Sodium 4-Nitrophenolate	y=8200.8x+21079	0.9995	0.36	1.21	22.22
24	2,4-D	y=1812.3x-2864.4	0.9997	0.48	1.59	-50.67
25	Dimethoate	y=48961x+85638	0.9993	0.18	0.59	155.82
26	Acetamiprid	y=56755x+108562	0.9993	0.15	0.50	132.05
27	Carbendazim	y=139898x+38035	0.9999	0.17	0.58	165.93
28	Thiacloprid	y=96867x+183064	0.9993	0.18	0.61	142.93
29	Trinexapac-Ethyl	y=10889x+8364.3	0.9998	1.04	3.45	163.84
30	Tricyclazole	y=51689x+4964.5	0.9994	0.55	1.85	-100.00
31	Indolbutyric Acid	y=6887.5x-2672.2	0.9999	1.33	4.43	-100.00
32	6-Benzylaminopurine	y=133006x+40644	0.9995	0.59	1.98	281.17
33	Thidiazuron	y=17906x-3955.9	0.9998	0.23	0.76	8.04
34	Dichlorvos	y=39787x+2562.2	0.9999	0.30	1.01	37.68
35	Carbofuran	y=130099x-26323	0.9999	0.11	0.38	69.61
36	Carbaryl	y=33092x-8776.1	0.9998	0.24	0.78	546.23
37	Pirimicarb	y=138229x-26643	0.9998	0.11	0.36	27.87
38	Forchlorfenuron	y=38305x-9752.6	0.9997	0.29	0.98	23.31
39	RH-5849	y=23292x+30796	0.9991	0.18	0.59	38.13
40	Isopropyl	y=94431x+24479	0.9999	0.23	0.76	26.65
41	Atrazine	y=51461x+50072	0.9995	0.29	0.95	18.56
42	Imidan	y=98202x-9327.4	1	0.12	0.39	-21.55
43	Chlorantraniliprole	y=22285x+4982.3	0.9999	0.21	0.71	11.81
44	Clomazone	y=216809x-179760	0.9995	0.18	0.59	24.76
45	Azoxystrobin	y=353301x-366207	0.9999	0.34	1.13	77.94
46	Methyl Jasmonate	y=15695x+4115.7	0.9998	0.58	1.93	16.45

47	Pyrimethanil	y=26105x-11245	0.9997	0.19	0.64	18.18
48	Fenamidone	y=63277x-22054	0.9997	0.25	0.83	-12.46
49	Boscalid	y=43899x+14528	0.9998	0.87	2.91	3.70
50	Dimethomorph	y=63461x+32882	0.9997	0.38	1.27	-14.62
51	Mandipropamid	y=162904x-148884	0.9996	0.22	0.72	-0.70
52	Isoprothiolane	y=228197x+18351	0.9999	0.18	0.61	26.62
53	Fluopicolide	y=64493x+61888	0.9995	0.15	0.49	12.23
54	Paclobutrazol	y=37679x+56533	0.9994	0.78	2.59	-3.53
55	Uniconazole	y=26410x+33743	0.9996	0.38	1.27	-0.96
56	Malathion	y=81948x+702195	0.9992	0.37	1.23	11.76
57	Orysastrobin	y=199043x-176598	0.9995	0.17	0.56	0.07
58	Benthiavalicarb-Isopropyl	y=76225x+42575	0.9995	0.39	1.31	-6.82
59	Methoxyfenozide	y=35307x+37728	0.9999	0.94	3.14	12.65
60	Triadimefon	y=38788x+9201.3	0.9999	0.87	2.89	1.24
61	Tiadinil	y=10779x+8399.6	0.996	0.15	0.51	-7.64
62	Cyproconazole	y=19663x++22588	0.9994	0.22	0.74	2.19
63	Triazophos	y=404253x-433594	0.9995	1.00	3.32	1.40
64	Fenhexamid	y=23504x-9706.8	0.9997	0.24	0.78	-10.99
65	Chromafenozide	y=93934x+1903.8	0.9999	0.48	1.59	11.43
66	Tetrachlorantraniliprole	y=4213.7x-1449.5	0.9992	0.29	0.95	-13.88
67	Spirotetramat	y=66194x-54820	0.9999	0.17	0.57	-29.08
68	Epoxiconazole	y=79125x+43634	0.9994	0.19	0.64	-12.42
69	Furan Tebufenozide	y=22802x+10332	0.9994	0.21	0.71	4.36
70	Diclocymet	y=8666.8x+4298.4	0.9995	0.07	0.25	-13.42
71	Alachlor	y=43013x+22425	0.9993	0.27	0.89	17.17
72	Tetraconazole	y=39710x+4677.2	0.9994	0.07	0.24	-0.15

73	Cyazofamid	y=60255x+62571	0.9997	0.05	0.16	0.53
74	Fenoxanil	y=90810x-753.98	0.9991	0.10	0.33	15.66
75	Picoxystrobin	y=180391x-150736	0.9996	0.05	0.16	33.77
76	Flusilazole	y=40461x+35688	0.9994	0.14	0.47	11.99
77	Kresoxim-Methyl	y=30858x-2991.5	0.9998	0.34	1.12	-100
78	Fipronil	y=35776x+35770	0.9993	0.05	0.18	-31.2
79	Diazinon	y=159203x-180621	0.9993	0.10	0.33	21.52
80	Brassinolide	y=1187.9x+1377	0.9993	3.84	12.80	15.98
81	Tebuconazole	y=58562x+7110.4	0.9998	0.21	0.70	-2.72
82	Pentrimazole	y=71050x+33395	0.9997	0.15	0.19	18.66
83	Pythiamin	y=215166x+26355	0.9993	0.16	0.54	27.11
84	Propiconazole	y=33052x+23629	0.9993	0.25	0.82	-5.92
85	Pyraoxystrobin	y=566311x+673584	0.9993	0.16	0.53	13.63
86	Pyraclostrobin	y=51345x+84725	0.9996	0.06	0.20	13.05
87	Phoxim	y=17911x+14074	0.9992	0.21	0.70	25.56
88	Cyflufenamid	y=113712x-124712	0.9995	0.24	0.79	20.33
89	Diniconazole	y=29278x+1139.5	0.9999	0.26	0.87	-9.06
90	Ametoctradin	y=187542x-70967	0.9991	0.20	0.66	46.01
91	Indoxacarb	y=9975x+14010	0.9993	0.21	0.71	-15.44
92	Pretilachlor	y=424448x-365561	0.9996	0.08	0.27	43.98
93	Difenoconazole	y=93030x-28532	0.9998	0.28	0.94	-5.93
94	Trifloxystrobin	y=241872x-199253	0.9996	0.13	0.45	33.85
95	Profenofos	y=55466x-13390	0.9999	0.06	0.21	12.32
96	Enestroburin	y=92901x-52970	0.9997	0.23	0.76	5.70
97	Coumoxystrobin	y=59606x+3415	0.9992	0.62	2.07	2.33
98	Buprofezin	y=141664x-104352	0.9996	0.18	0.60	19.69

99	Emamectin Benzoate	y=107366x-57134	0.9998	0.02	0.07	-10.25
100	Fenpropathrin	y=9061.2x-6411.7	0.999	0.70	2.33	45.38
101	Chlorpyrifos	y=39001x+3275.5	0.9991	0.07	0.24	6.05
102	Hexythiazox	y=62569x-74023	0.9994	0.04	0.13	2.69
103	Propargite	y=106538x-76302	0.9998	0.06	0.21	2.77
104	Spirodiclofen	y=21120x+8852.3	0.9994	0.16	0.54	0.10
105	Pyridaben	y=193825x-171370	0.9996	0.27	0.89	17.41
106	Carbosulfan	y=97355x-6575.5	0.9997	0.05	0.18	2.59
107	Etofenprox	y=68716x+84214	0.9997	0.16	0.52	13.56
108	Bifenthrin	y=13120x+11077	0.9998	0.12	0.39	-2.79

Table S4. Calibration curves, R^2, LOD, LOQ, and matrix effects for each target pesticide in *Chrysanthemum Morifolium* Ramat.

No.	Pesticide	Calibration curves	R^2	LOD (μg/kg)	LOQ (μg/kg)	ME (%)
1	Betaine	y=726.23x+60235	0.9998	0.36	1.19	-94.93
2	Acephate	y=36270x+22887	0.9997	0.91	3.05	-71.70
3	Omethoate	y=13561x+1029.9	0.9996	0.95	3.16	-71.48
4	3-Indoleacetic acid	y=5717.7x+2960.2	0.9996	0.65	2.16	-16.22
5	Aldicarb sulfoxide	y=4032.7x-1972.7	0.9995	0.43	1.42	-67.31
6	Dinotefuran	y=9475.3x-1336.8	0.9992	2.08	6.93	-34.83
7	Propamocarb	y=13589x-5615.1	0.9998	0.70	2.32	-25.54
8	Aldicarb sulfone	y=25941x+13075	0.9993	1.18	3.94	-32.71
9	Methomyl	y=8667.9x-11044	0.9993	0.13	0.43	-37.69
10	Deisopropylatrazine	y=1871.7x+471.17	0.9994	1.09	3.64	-69.99
11	Thiamethoxam	y=21368x-14945	0.9998	0.23	0.78	-42.71
12	Gibberellic acid	y=707.07x+1474.3	0.9997	3.75	12.48	-13.93
13	Abscisic Acid	y=9264.2x-7529.1	0.9998	0.43	1.44	40.12
14	Pyrazosulfuron-Ethyl	y=23433x-25500	0.9993	0.19	0.62	30.28
15	Cycloxaprid	y=7388.1x-3174.5	0.9994	0.72	2.41	-68.74
16	Clothianidin	y=5314.7x-2705.9	0.9994	0.25	0.82	-51.04
17	Imidacloprid	y=7448.3x-8157.8	0.9991	0.92	3.08	-62.80
18	Trichlorfon	y=4221.2x+8648.3	0.9985	0.42	1.39	-61.25
19	3-Hydroxycarbofuran	y=3232.4x-2322.9	0.9991	1.07	3.56	-62.38
20	6-Kinetin	y=10435x+30209	0.9992	1.08	3.59	-87.67
21	Sodium 5-Nitroguaiacolate	y=1545.3x+1351.8	0.9997	2.09	6.98	-4.85
22	Sodium 2-Nitrophenolate	y=44445.x+322367	0.9985	0.25	0.84	-45.16

23	Sodium 4-Nitrophenolate	y=3990.4x+19687	0.9997	0.25	0.82	-47.12
24	2,4-D	y=2842x+243.81	0.9997	0.56	1.87	179.01
25	Dimethoate	y=13672x-9121.4	0.9994	0.18	0.60	-69.71
26	Acetamiprid	y=17310x-13376	0.9992	0.18	0.59	-65.19
27	Carbendazim	y=27370x-6627.7	0.9991	0.16	0.53	-78.45
28	Thiacloprid	y=24626x-22169	0.9993	0.47	1.55	-70.87
29	Trinexapac-Ethyl	y=5544.6x-1811.7	0.9994	1.33	4.43	-52.58
30	Tricyclazole	y=11800x-14282	0.9992	0.22	0.74	-75.79
31	Indolbutyric Acid	y=2722.9x+59756	0.9988	1.13	3.75	-54.64
32	6-Benzylaminopurine	y=39910x-7489.7	0.9993	0.70	2.32	-65.31
33	Thidiazuron	y=7508.7x-5155.1	0.9994	0.16	0.55	-53.53
34	Dichlorvos	y=11239x+187577	0.9809	0.96	3.22	-67.63
35	Carbofuran	y=2235x-26782	0.999	0.27	0.91	-87.31
36	Carbaryl	y=5090.4x+43300	0.9984	0.96	3.22	-81.81
37	Pirimicarb	y=32304x-36643	0.9992	0.11	0.36	-75.26
38	Forchlorfenuron	y=13786x-3115.8	0.9998	0.47	1.56	-54.85
39	RH-5849	y=1368.9x-1990.2	0.9998	0.97	3.22	-92.33
40	Isopropyl	y=19338x-9372.1	0.9998	0.58	1.92	-76.81
41	Atrazine	y=10208x+7702.3	0.9994	0.67	2.25	-77.58
42	Imidan	y=37782x-26579	0.9997	0.18	0.61	-63.00
43	Chlorantraniliprole	y=7751.7x+9274.5	0.9993	0.27	0.89	-58.59
44	Clomazone	y=45107x-34626	0.9999	1.17	3.91	-74.10
45	Azoxystrobin	y=143993x-321227	0.9987	0.08	0.25	-54.25
46	Methyl Jasmonate	y=4818x+58990	0.9997	0.58	1.93	-61.17
47	Pyrimethanil	y=7002.5x-2083.7	0.9991	0.53	1.75	-70.24
48	Fenamidone	y=33900x-41349	0.9992	0.95	3.17	-35.65

49	Boscalid	y=18252x-11997	0.9992	0.47	1.57	-48.14
50	Dimethomorph	y=30490x-16695	0.9996	0.57	1.89	-41.05
51	Mandipropamid	y=75507x-69922	0.9998	0.16	0.53	-47.71
52	Isoprothiolane	y=60724x+99631	0.9998	0.25	0.85	-69.05
53	Fluopicolide	y=20300x-10945	0.9996	0.29	0.97	-63.18
54	Paclobutrazol	y=155133x+4100.8	0.9994	1.03	3.43	-51.64
55	Uniconazole	y=12071x+3608.2	0.9999	0.75	2.49	-46.54
56	Malathion	y=23456x+52122	0.9998	0.91	3.05	-67.74
57	Orysastrobin	y=102107x-99470	0.9992	0.37	1.23	-49.17
58	Benthiavalicarb-Isopropyl	y=30566x-26390	0.9995	0.35	1.18	-55.65
59	Methoxyfenozide	y=11828x-8080	0.9998	0.83	2.78	-62.79
60	Triadimefon	y=14955x-5891.4	0.9998	0.78	2.61	-53.40
61	Tiadinil	y=10299x-4292.9	0.9995	0.12	0.40	-3.13
62	Cyproconazole	y=8602.3x+438.1	0.9998	0.88	2.93	-46.30
63	Triazophos	y=151884x-121530	0.9998	0.26	0.87	-56.68
64	Fenhexamid	y=12296x-1990.8	0.9997	0.38	1.28	-43.39
65	Chromafenozide	y=39951x-14378	0.9999	0.52	1.75	-50.84
66	Tetrachlorantraniliprole	y=2068x+132.66	0.9999	0.55	1.85	-45.06
67	Spirotetramat	y=67668x43968	0.9997	0.18	0.59	-100.00
68	Epoxiconazole	y=3403x+349538	0.9999	0.48	1.61	-48.35
69	Furan Tebufenozide	y=11266x-5811.3	0.9996	0.83	2.77	-50.92
70	Diclocymet	y=8513.5x-4216.1	0.9997	0.11	0.36	3.95
71	Alachlor	y=13771x+27196	0.9999	0.96	3.19	-66.31
72	Tetraconazole	y=13260x+5025.5	0.9995	0.72	2.40	-59.22
73	Cyazofamid	y=19722x-8365.2	0.9999	0.26	0.88	-67.73
74	Fenoxanil	y=32903x-26110	0.9998	0.22	0.75	-55.86

75	Picoxystrobin	y=64210x-38207	0.9999	0.24	0.78	-60.41
76	Flusilazole	y=13871x+840296	0.9996	0.24	0.81	-58.79
77	Kresoxim-Methyl	y=7681.4x+6.4705	0.9997	0.40	1.33	-71.75
78	Fipronil	y=40572x-17310	0.9998	0.14	0.47	3.96
79	Oxyfluorfen	y=40854x+136.24	0.9993	0.63	2.09	-70.04
80	Diazinon	y=1676.1x+1386.8	0.9998	0.64	2.13	46.49
81	Brassinolide	y=25252x+11157	0.9995	3.70	12.34	-47.26
82	Tebuconazole	y=23275x-10134	0.9999	0.99	3.29	-59.46
83	Pentrimazole	y=71803x-80580	0.9997	0.11	0.37	-62.26
84	Pythiamin	y=15186x+11952	0.9998	0.77	2.57	-45.78
85	Propiconazole	y=263936x-239088	0.9994	0.23	0.78	-57.90
86	Pyraoxystrobin	y=29130x+49144	0.9998	0.10	0.35	-51.06
87	Pyraclostrobin	y=19886x+15418	0.9999	0.94	3.14	-68.11
88	Phoxim	y=27085x-21454	0.9992	0.25	0.85	-67.32
89	Cyflufenamid	y=12004x-532.9	0.9992	0.84	2.81	-53.60
90	Diniconazole	y=77551x-59122	0.9995	0.10	0.32	-53.29
91	Ametoctradin	y=6377.5x-2735.6	0.9995	0.39	1.31	-30.55
92	Indoxacarb	y=132496x+88180	0.9992	0.46	1.54	-63.79
93	Pretilachlor	y=42841x+11449	0.9992	0.21	0.69	-46.45
94	Difenoconazole	y=72245x-60902	0.9993	0.11	0.35	-65.69
95	Trifloxystrobin	y=22319x-26283	0.9992	0.62	2.08	-50.72
96	Profenofos	y=42641x-2744.3	0.9993	0.37	1.24	-50.48
97	Enestroburin	y=37995x-14482	0.9996	0.97	3.24	60.24
98	Coumoxystrobin	y=54778x-32580	0.9994	0.14	0.45	-54.33
99	Buprofezin	y=94661x-13474	0.9996	0.01	0.04	-25.96
100	Emamectin Benzoate	y=3088.6x+8764.5	0.9997	0.88	2.92	-49.27

101	Fenpropathrin	y=14372x+26635	0.9993	0.33	1.11	-57.02
102	Chlorpyrifos	y=21852x+4394.7	0.9998	0.08	0.27	-58.02
103	Hexythiazox	y=35783x-8279.7	0.9993	0.18	0.59	-59.95
104	Propargite	y=9681.7x+7988.3	0.9999	0.46	1.54	-55.92
105	Spirodiclofen	y=80896x-62508	0.9997	0.21	0.71	-55.99
106	Pyridaben	y=35511x+45199	0.9998	0.56	1.85	-64.09
107	Carbosulfan	y=36803x-12237	0.9997	0.19	0.64	-55.28
108	Etofenprox	y=4386.2x+5940.8	0.9993	0.08	0.27	-52.99

Table S5. Calibration curves, R^2, LOD, LOQ, and matrix effects for each target pesticide in *Dendrobium officinale* Kimura et Migo.

No.	Pesticide	Calibration curves	R^2	LOD (μg/kg)	LOQ (μg/kg)	ME (%)
1	Betaine	y=2008.7x+4824.5	0.9987	0.09	0.31	-87.74
2	Acephate	y=7234.7x+22622	0.9986	0.52	1.75	-71.59
3	Omethoate	y=13695x+22012	0.9992	0.10	0.34	-66.89
4	3-Indoleacetic acid	y=8787.7x+94389	0.9994	0.10	0.34	-6.5
5	Aldicarb sulfoxide	y=4532.6x-5465.8	0.9990	0.43	1.43	-63.84
6	Dinotefuran	y=14769x+27405	0.9995	0.45	1.51	-16.07
7	Propamocarb	y=9391.3x+126922	0.9987	0.09	0.30	-51.04
8	Aldicarb sulfone	y=43431x+65890	0.9995	0.34	1.24	-2.09
9	Methomyl	y=14387x+7626.6	0.9997	0.17	0.55	-12.75
10	Deisopropylatrazine	y=4305.6x+4573.8	0.999	0.45	1.50	-42.87
11	Thiamethoxam	y=44661x+4691.7	0.9994	0.06	0.19	-2.46
12	Gibberellic acid	y=993.55x+1181.1	0.9991	1.94	6.46	30.40
13	Abscisic Acid	y=2485.2x+33857	0.9995	0.13	0.43	27.21
14	Pyrazosulfuron-Ethyl	y=35151x+22518	0.9997	0.16	0.545	49.19
15	Cycloxaprid	y=26611x+21221	0.9995	0.40	1.34	-13.49
16	Clothianidin	y=10259x+10700	0.9995	0.27	0.89	-19.59
17	Imidacloprid	y=19032x+496283	0.9996	0.20	0.66	-100
18	Trichlorfon	y=12598x+23142	0.9996	0.36	1.21	-11.96
19	3-Hydroxycarbofuran	y=7297.7x+8089.9	0.9991	0.26	0.86	-40.94
20	6-Kinetin	y=27231x+26972	0.9998	1.16	3.87	-70.61
21	Sodium 5-Nitroguaiacolate	y=1358.1x+5206.7	0.9991	3.87	12.90	1.34
22	Sodium 2-Nitrophenolate	y=6716.4x+54049	0.999	0.14	0.46	-13.14

23	Sodium 4-Nitrophenolate	y=6324.7x+50268	0.9997	0.28	0.95	-17.15
24	2,4-D	y=2693.3x+297.97	0.9994	0.49	1.64	5.15
25	Dimethoate	y=32484x-775.7	0.9994	0.04	0.12	-43.32
26	Acetamiprid	y=39630x-573.08	0.9992	0.07	0.25	-42.94
27	Carbendazim	y=56976x+47771	0.9999	0.18	0.59	-100
28	Thiacloprid	y=49912x+23108	0.9999	0.26	0.87	-53.63
29	Trinexapac-Ethyl	y=13289x+1464.8	0.9999	0.95	3.17	-16.15
30	Tricyclazole	y=24513x+2994.6	0.9999	0.38	1.27	-53.92
31	Indolbutyric Acid	y=4875.6x+5036.5	0.9996	1.03	3.44	-19.40
32	6-Benzylaminopurine	y=56841x+19541	0.9999	1.01	3.37	-49.22
33	Thidiazuron	y=14120x+11698	0.9997	0.22	0.74	-13.08
34	Dichlorvos	y=22705x+21038	0.9998	0.98	3.26	-34.84
35	Carbofuran	y=95026x+32237	0.9998	0.11	0.37	-33.92
36	Carbaryl	y=20614x+12655	0.9998	0.48	1.59	-38.62
37	Pirimicarb	y=116308x+69339	0.9996	0.12	0.39	-24.39
38	Forchlorfenuron	y=28551x+24131	0.9995	0.31	1.02	-15.87
39	RH-5849	y=20877x+10985	0.9997	0.27	0.89	16.11
40	Isopropyl	y=71656x+68057	0.9995	0.22	0.75	-29.09
41	Atrazine	y=28916x+34972	0.9995	0.26	0.86	-33.66
42	Imidan	y=109490x+17767	0.9998	0.04	0.13	-15.19
43	Chlorantraniliprole	y=21856x+17700	0.9996	0.11	0.36	-2.24
44	Clomazone	y=113319x-11497	1	0.19	0.64	-45.31
45	Azoxystrobin	y=305991x-233460	0.9999	0.18	0.61	-8.2
46	Methyl Jasmonate	y=11584x+26911	0.9994	0.10	0.32	-9.66
47	Pyrimethanil	y=21915x+13077	0.9998	0.09	0.31	-18.7
48	Fenamidone	y=64819x+24224	0.9996	0.12	0.39	4.73

49	Boscalid	y=39405x+14744	0.9997	0.10	0.32	1.72
50	Dimethomorph	y=60845x+103080	0.9995	0.55	1.83	7.86
51	Mandipropamid	y=149917x+851119	0.9995	0.14	0.48	-4.58
52	Isoprothiolane	y=190028x+159898	0.9997	0.12	0.41	-13.93
53	Fluopicolide	y=50415x+147655	0.9986	0.09	0.30	-12.01
54	Paclobutrazol	y=36433x+76453	0.9997	0.20	0.68	-1.28
55	Uniconazole	y=25250x+42624	0.9996	0.16	0.54	-9.01
56	Malathion	y=73056x+756749	0.9996	0.21	0.39	-8.24
57	Orysastrobin	y=178194x+141730	0.9995	0.42	1.41	-11.03
58	Benthiavalicarb-Isopropyl	y=66069x+68028	0.9998	0.10	0.34	-5.11
59	Methoxyfenozide	y=33275x+65546	0.9994	0.21	0.69	-11.21
60	Triadimefon	y=40481x+18441	0.9996	0.55	1.84	4.78
61	Tiadinil	y=11855x+24701	0.9993	0.10	0.34	20.22
62	Cyproconazole	y=20006x+21949	0.9997	0.11	0.38	-7.56
63	Triazophos	y=369262x+18469	0.9999	0.13	0.46	12.36
64	Fenhexamid	y=22483x+14565	0.9994	0.12	0.39	0.63
65	Chromafenozide	y=7913x+1508.9	0.9993	0.06	0.19	-12.02
66	Tetrachlorantraniliprole	y=4457.1x+3241	0.9999	0.12	0.41	-8.24
67	Spirotetramat	y=70767x-12432	0.9994	0.10	0.33	22.02
68	Epoxiconazole	y=77647x+117208	0.9994	0.54	1.80	0.47
69	Furan Tebufenozide	y=17951x+11947	0.9997	0.26	0.87	-100
70	Diclocymet	y=8859.4x+12537	0.9997	0.12	0.39	14.27
71	Alachlor	y=35566x+21093	0.9996	0.42	1.400.1	-16.18
72	Tetraconazole	y=34673x+18611	0.9997	0.27	0.89	-6.62
73	Cyazofamid	y=55531x+40511	0.9997	0.26	0.87	-12.67
74	Fenoxanil	y=79786x+36360	0.9997	0.11	0.36	-12.59

75	Picoxystrobin	y=113573x+99771	0.9995	0.04	0.13	-17.97
76	Flusilazole	y=39595x+18697	0.9997	0.17	0.56	-8.01
77	Kresoxim-Methyl	y=23354x+250776	0.9995	0.29	0.98	-23.12
78	Fipronil	y=49547x+69488	0.9993	0.06	0.22	46.26
79	Diazinon	y=112558x+74648	0.9997	0.10	0.33	-23.06
80	Brassinolide	y=978.14x+4327.4	0.999	3.21	10.70	-6.94
81	Tebuconazole	y=41062x+912705	0.9992	0.04	0.14	-0.92
82	Pentrimazole	y=55749x+23564	0.9995	0.18	0.60	-14.01
83	Pythiamin	y=185869x-2518.1	0.9993	0.18	0.59	-13.23
84	Propiconazole	y=30680x+24329	0.9998	0.12	0.42	4.46
85	Pyraoxystrobin	y=561606x+749517	0.9997	0.51	0.49	-4.40
86	Pyraclostrobin	y=50002x+969842	0.998	0.05	0.16	-18.42
87	Phoxim	y=42844x+185293	0.9974	0.16	0.53	-11.88
88	Cyflufenamid	y=64361x+48359	0.9991	0.13	0.44	-7.89
89	Diniconazole	y=29690x+13026	0.9997	0.22	0.73	2.29
90	Ametoctradin	y=146300x+106163	0.9995	0.19	0.63	-18.13
91	Indoxacarb	y=11818x+11625	0.9999	0.17	0.58	13.96
92	Pretilachlor	y=366634x+110050	0.9998	0.80	2.66	-10.18
93	Difenoconazole	y=99221x+553000	0.9993	0.04	0.15	11.50
94	Trifloxystrobin	y=186495x+105698	0.9996	0.13	0.44	-13.98
95	Profenofos	y=49890-6532.5	0.9994	0.45	1.52	-4.98
96	Enestroburin	y=82926x+73350	0.9993	0.08	0.25	-5.47
97	Coumoxystrobin	y=52020x+84700	0.9995	0.39	1.32	-8.34
98	Buprofezin	y=112379x+150781	0.9992	0.18	0.61	-15.82
99	Emamectin Benzoate	y=98415x+86666	0.9997	0.111	0.38	5.06
100	Fenpropathrin	y=5041.5x+24912	0.9996	0.58	1.94	-30.97

101	Chlorpyrifos	y=32040x++158091	0.9996	0.08	0.26	-14.76
102	Hexythiazox	y=57018x+17552	0.9996	0.12	0.38	-2.82
103	Propargite	y=110744x-9268.2	0.9997	0.08	0.28	-2.36
104	Spirodiclofen	y=21537x+8722.6	0.9999	0.27	0.90	0.32
105	Pyridaben	y=170674x+54485	0.9995	0.06	0.21	-6.16
106	Carbosulfan	y=98378x+86087	0.9997	0.05	0.28	-3.35
107	Etofenprox	y=63424x+63045	0.9993	0.10	0.32	-10.81
108	Bifenthrin	y=12713x+2481.7	0.9998	0.48	1.59	26.80

Table S6. Average recoveries and RSD of 108 pesticides in *Fritillaria thunbergii* Miq, *Chrysanthemum Morifolium* Ramat, and *Dendrobium officinale* Kimura et Migo.

No.	Pesticides	***Fritillaria thunbergii* Miq**					***Chrysanthemum Morifolium* Ramat**					***Dendrobium officinale* Kimura et Migo**				
		Mean recovery (%)			Intra-day RSD (%)	Inter-day RSD (%)	Mean recovery (%)			Intra-day RSD (%)	Inter-day RSD (%)	Mean recovery (%)			Intra-day RSD (%)	Inter-day RSD (%)
		20 µg/kg	50 µg/kg	100 µg/kg			20 µg/kg	50 µg/kg	100 µg/kg			20 µg/kg	50 µg/kg	100 µg/kg		
1	Betaine	16.1	23.9	17.52	5.07-6.39	5.43	61.59	62.62	69.93	3.65-12.83	4.05	82.36	85.26	73.99	0.63-2.63	2.53
2	Acephate	86.08	77.98	75.94	1.80-3.99	5.44	84.36	71.79	75.06	2.36-6.89	9.92	75.21	76.36	76.28	0.74-2.24	9.65
3	Omethoate	76.69	74.56	80.23	2.61-4.37	4.44	80.66	83.4	86.46	1.77-8.31	7.83	78.16	70.17	78.82	2.72-4.09	8.54
4	3-Indoleacetic Acid	92.49	97.38	93.9	2.93-6.13	6.54	107.13	97.78	104.68	1.57-1.91	6.67	106.8	97.06	93.85	2.06-3.30	3.14
5	Aldicarb sulfoxide	76.08	80.8	88.47	3.3-4.44	4.47	72.81	72.06	81.41	4.35-7.42	5.93	81.09	79.77	83.73	2.28-3.95	8.12
6	Dinotefuran	80.42	87.65	96.58	2.03-7.37	5.83	79.93	82.12	83.25	2.12-5.5	5.92	94.79	88.81	92.25	1.74-2.18	10.16
7	Propamocarb	74.61	72.43	74.62	2.91-5.45	8.61	69.45	61.21	68.59	2.57-3.19	4.35	77.92	72.26	79.56	2.25-3.58	2.72
8	Aldicarb Sulfone	90.15	109.95	94.59	2.23-4.77	4.14	96.25	91.54	101.24	1.77-2.04	2.87	89.89	89.26	92.65	0.75-3.38	4.28
9	Methomyl	89.79	99.19	92.14	1.98-4.32	4.38	95.25	92.69	96.18	1.85-4.45	3.71	94.79	88.81	92.25	1.74-2.18	3.62
10	Deisopropylatrazine	87.74	93.42	93.33	1.13-5.73	5.05	80.39	93.65	108.28	7.38-8.18	7.9	92	90.25	89.11	2.76-6.35	7.43
11	Thiamethoxam	92.92	94.57	93.37	1.45-2.49	3.14	94.67	90.45	99.17	1.73-5.45	6.31	97.55	88	88.65	0.8-2.59	2.38
12	Gibberellic Acid	91.03	73.7	79.05	4.15-6.65	4.4	83.58	77.2	82.52	2.16-6.93	10.01	83.64	87.85	83.87	3.08-3.68	10.74
13	Abscisic Acid	94.2	95.66	97.27	1.44-2.77	2.51	110.01	111.12	110.1	1.11-5.6	12.36	111.52	107.9	96.53	2.01-7.83	9.5
14	Pyrazosulfuron-Ethyl	81.73	99.98	92.25	2.77-5.08	8.39	104.72	95.69	93.34	1.74-3.27	2.98	104.85	94.03	92.8	2.26-3.22	2.97
15	Cycloxaprid	94.54	95.74	88.18	1.70-6.65	5.06	70.54	72.49	78.29	6.27-8.33	6.08	73.26	74.98	78.52	1.59-2.27	3.22
16	Clothianidin	92.06	93.59	91.52	3.88-6.19	4.3	87.24	91.62	95.94	2.86-4.69	6.6	97.8	87.15	90.22	3.73-4.21	3.74
17	Imidacloprid	94.86	97.45	94.5	3.40-4.08	7.85	85.43	93.31	94.93	2.26-6.74	5.72	104.43	105	101.94	1.31-2.65	1.19
18	Trichlorfon	87.13	89.24	91.76	2.33-3.62	2.82	90.7	89.17	103.99	3.25-14.21	5.56	96.25	98.31	95.5	1.15-3.37	11.91

19	3-Hydroxycarbofuran	97.35	93.96	92.14	4.96-5.72	5.67	86.65	86.42	87.72	2.75-7.06	9.07	96.33	98.36	97.23	1.99-5.26	4.15
20	6-Kinetin	44.3	45.07	35.35	3.53-16.02	11.57	61.53	63.93	61.47	1.44-11.74	6.68	49.16	34.66	35.51	0.13-6.96	5.58
21	Sodium 5-Nitroguaiacolate	103.8	95.86	98.68	5.43-12.16	3.76	108.14	103.03	104.16	2.07-6.84	11.01	92.57	80.52	96.3	2.63-8.13	4.6
22	Sodium 2-Nitrophenolate	90.58	95.92	93.31	1.70-4.81	6.38	108.66	100.3	106.49	1.61-2.35	1.87	92.42	96.97	94.53	3.58-6.93	6.64
23	Sodium 4-Nitrophenolate	91.88	88.04	93.62	1.56-3.80	5.14	107.39	104.29	106.67	2.69-2.94	1.94	99.63	94.23	95.71	1.65-7.11	6.78
24	2,4-D	80.37	78.3	79.86	5.21-9.00	4.61	95.28	91.73	94.86	2.29-5.03	6.77	82.4	77.71	77.44	4.87-12.58	9.85
25	Dimethoate	103.49	101.07	92.7	2.97-4.40	4.53	98.92	93.09	97.76	0.46-8.61	5.43	109.12	96.27	97.04	1.52-3.81	4.85
26	Acetamiprid	97.53	95.85	94.3	0.81-5.40	4.95	102.86	87.62	94.5	3.38-5.7	3.02	106.92	97.67	97.79	1.83-3.63	3.5
27	Carbendazim	75.25	78.86	72.94	2.34-4.22	2.76	78.52	77.45	81.83	1.65-4.73	6.89	79.42	78.83	74.11	3.85-4.83	6.21
28	Thiacloprid	92.76	98.71	96.75	1.11-4.16	4.22	106.43	94.41	101.13	1.64-7.77	3.06	101.63	90.79	92.63	1.16-4.34	5.38
29	Trinexapac-Ethyl	95.84	101.16	95.5	4.66-16.82	11.61	94.55	95.68	96.09	1.87-2.89	5.21	93.55	94.98	94.84	2.51-8.98	8.71
30	Tricyclazole	78.12	83.84	75.71	3.74-11.11	2.03	88.75	75.32	87.02	1.9-7.74	5.53	75.58	70.88	76.45	2.12-3.88	4.26
31	Indolbutyric Acid	79.86	90.36	93.71	4.73-8.73	6.69	88.03	90.27	95.32	3.03-5.61	6.78	87.93	86.98	84.24	6.37-9.90	7.76
32	6-Benzylaminopurine	17.82	26.55	27.55	6.48-8.92	6.48	68.97	63.11	69.48	1.76-7.79	11.79	55.13	45.54	48.73	2.44-5.38	5.55
33	Thidiazuron	52.47	46.87	41	1.60-8.62	8.62	109.7	87.15	93.93	1.96-11.35	5.36	66.92	63.95	63.37	3.35-3.95	5.69
34	Dichlorvos	105.71	97.16	96.38	1.38-7.02	1.21	110.3	96.27	100.19	1.56-3.16	1.73	105.81	93.85	94.46	2.45-6.65	6.87
35	Carbofuran	89.04	101.18	107.93	2.30-7.76	5.14	109.34	100.3	103.91	1.33-3.8	3.74	107.12	97.99	100.09	2.13-2.28	1.87
36	Carbaryl	93.29	89.27	87.96	4.46-10.05	8.73	109.19	86.36	90.23	6.37-10.92	6.99	98.13	89.78	93.1	2.25-6.31	6.13
37	Pirimicarb	92.93	103.46	96.71	1.75-4.73	3.63	108.12	92.95	102.29	1.47-3.17	1.53	98	90.82	92.43	2.06-2.54	2.33
38	Forchlorfenuron	53.71	52.82	47.43	2.69-4.84	6.39	100.37	91.54	100.21	4.63-11.57	5.92	78.26	74.01	72.68	2.55-3.53	3.9
39	RH-5849	91.99	106.8	96.79	1.80-4.38	4.34	78.94	80.75	96.05	3.75-5.79	5.53	98.3	93.54	93.02	2.95-5.15	4.18
40	Isopropyl	91.87	108.06	95.42	2.23-4.84	5.38	105.46	94.38	98.53	1.23-7.31	2.19	101.48	92.79	93.48	2.13-3.01	2.66
41	Atrazine	98.22	108.7	93.9	1.69-4.65	2.22	97.89	97.55	103.75	2.64-4.18	9.76	99.67	92.06	92.65	2.39-2.96	4.04

42	Imidan	97.3	96.86	97.62	2.52-18.37	2.15	94.77	94.43	97.59	1.39-2.87	2.7	114.41	93.32	94.04	2.87-3.68	2.35
43	Chlorantraniliprole	109.49	104.61	97.62	3.25-8.00	2.89	79.13	92.36	97.45	2.4-3.08	6.21	103.56	91.86	93.46	1.26-2.88	3.56
44	Clomazone	87.71	101.78	101.12	2.62-4.15	6.25	96.09	96.24	99.42	0.7-1.68	1.92	102.24	91.86	94.14	1.52-2.37	2.29
45	Azoxystrobin	90.22	99.23	89.01	2.43-5.05	4.19	104.95	95.91	105.19	1.79-3.58	3.6	103.9	101.78	97.55	1.38-2.22	1.78
46	Methyl Jasmonate	91.78	98.06	92.58	1.92-6.24	4.75	108.52	98.08	97.9	1.78-4.49	3.35	97.61	94.62	94.14	4.10-6.57	5.48
47	Pyrimethanil	88.05	91.27	82.15	3.00-4.29	4.57	91.35	90.21	94.79	1.99-7.12	8.94	95.12	86.33	84.22	1.27-5.06	6.46
48	Fenamidone	93.69	89.46	89.85	1.46-2.33	4.07	94.31	95.75	96.77	0.76-3.78	6.08	96.36	93.12	94.39	1.88-3.87	4.49
49	Boscalid	96.97	97.31	92.36	0.83-2.90	2.64	100.31	96.46	99	0.8-5.66	5.1	107.68	92.99	94.17	1.45-9.42	7.36
50	Dimethomorph	97.29	99.39	94.19	0.27-2.48	2.12	97.58	92.09	97.79	2.75-4.42	4.95	109.04	104.24	100.76	1.55-3.20	1.59
51	Mandipropamid	95.9	97.12	97.25	1.27-3.88	3.02	97.95	95.66	95.9	1.02-3.5	1.64	104.51	93.63	96.52	1.46-3.43	1.75
52	Isoprothiolane	94.22	95.79	95.74	1.67-4.49	4.31	105.42	105.29	108.49	0.84-1.71	1.68	101.06	95.32	94.72	3.18-3.55	3.67
53	Fluopicolide	95.03	99.26	94.87	1.69-2.50	5.06	86.91	90.06	99.17	2.63-14.1	1.63	96.63	94.3	96.15	1.03-3.57	5.5
54	Paclobutrazol	93.05	94.98	97.44	1.20-2.22	5.65	83.34	90.92	95.82	1.27-11.84	5.03	109.14	96.16	92.94	3.34-3.9	3.04
55	Uniconazole	94.97	97.81	95.41	1.20-5.45	6.08	90.9	90.67	95.76	3.63-13.9	3.28	104.28	95.27	96.17	3.55-5.38	5.43
56	Malathion	93.52	92.19	93.9	2.23-3.30	2.9	98.3	96.23	95.67	1.19-2.26	5.05	94.73	91.13	96.89	2.61-4.94	2.53
57	Orysastrobin	95.54	95.3	98.24	0.86-2.65	2.18	98.63	97.37	98.12	0.81-1.56	1.24	105.67	92.64	96.36	2.43-2.83	3.22
58	Benthiavalicarb-Isopropyl	92.38	94.49	93.34	1.06-3.31	2.98	97.3	100.59	97.32	1.76-4.24	3.54	103.56	95.29	95.15	3.38-3.69	2.74
59	Methoxyfenozide	89.69	99.5	93.46	1.91-3.82	4.77	92.66	98.54	105.25	5.09-9.85	8.33	102.36	96.73	93.57	2.25-4.91	6.39
60	Triadimefon	83.19	91.79	89.16	1.32-6.04	5.04	77.03	97.44	96.67	1.02-7.58	6.96	112.92	93.33	93.09	2.33-6.06	9.31
61	Tiadinil	95.39	98.64	90.57	2.74-3.75	4.26	96.6	88.3	99.01	3.24-6.93	0.97	96.73	92	92.79	3.04-5.58	5.18
62	Cyproconazole	107.69	98.02	91.11	2.03-6.39	7.39	98.71	83.79	92.08	2.92-4.45	5.58	93.82	95.19	95.9	3.21-4.34	3.26
63	Triazophos	98.38	94.07	93.09	0.67-3.15	3.55	95.12	95.56	97.48	1.45-2.3	2.22	104.66	91.41	95.11	2.07-4.53	2.79
64	Fenhexamid	87.12	93.5	92.96	1.70-8.52	8.4	88.04	93.09	94.64	1.93-4.32	6.54	102.61	90.92	91.54	2.48-6.88	5.61
65	Chromafenozide	86.87	95.07	94.93	2.73-2.85	2.49	92.23	96.28	94.28	2.31-4.75	1.95	102.86	97.4	96.64	2.20-5.58	4.55
66	Tetrachlorantraniliprole	89.48	91.99	85.05	4.10-8.90	6.4	82.43	90.86	94.59	4.92-13.12	3.58	78.85	88.27	99.57	4.63-8.32	11.48

67	Spirotetramat	95.22	96.18	94.19	1.70-2.90	2.97	98.24	96.31	93.4	2.1-4.07	2.4	108.68	93.67	99.12	3.90-4.12	3.62
68	Epoxiconazole	89.8	89.16	93.07	1.91-4.84	7.71	93.53	89.56	96.54	1.84-6.41	2.25	99.05	94.64	93.22	3.04-3.94	4.34
69	Furan Tebufenozide	94.06	86.03	92.41	2.95-9.01	6.7	90.24	86.38	93.18	3.92-6.92	6.38	98.29	98.88	94.03	3.25-6.07	5.89
70	Diclocymet	92.55	101.57	92.19	3.17-6.51	6.27	95.98	91.95	100.13	1.28-4.72	6.82	112.26	94.33	97.98	2.75-6.95	11.66
71	Alachlor	87.51	92.3	93.69	0.93-4.75	3.57	92.68	95.194.03	94.71	1.03-4.61	7.56	101.43	94.22	96.34	3.53-6.26	6.59
72	Tetraconazole	109.4	107.52	97.02	2.42-6.32	4.96	97.69	90.37	98.32	2.58-4.74	2.39	96.73	92.33	94.46	2.76-3.90	4.78
73	Cyazofamid	94.15	106.25	97.01	2.92-3.94	3.94	101.93	94.51	96.5	1.05-6.84	4.42	97.56	91.24	93.9	2.46-3.85	3.98
74	Fenoxanil	94.82	96.34	91.59	2.21-4.95	4.95	99.18	95.37	97.96	0.59-5	1.64	98.56	95.71	95.05	2.72-5.08	3.57
75	Picoxystrobin	90.52	99.36	93.16	0.37-4.07	6.46	91.8	100.31	98.71	0.57-2.53	3.45	99.58	94.65	94.67	2.44-3.35	2.39
76	Flusilazole	96.34	93.5	91.87	3.78-4.69	3.5	105.68	97.99	97.15	0.59-2.38	0.68	94.13	93.08	92.4	2.72-6.32	5.39
77	Kresoxim-Methyl	99.34	97.71	96.69	1.37-2.18	4.73	89.3	92.79	94.91	2.77-4.58	5.27	102.39	98.6	96.1	2.55-2.85	3.18
78	Fipronil	93.12	97.38	96.23	1.44-3.77	6.85	95.22	98.69	97.41	1.65-2.52	2.29	100.09	96.81	92.14	3.25-4.32	2.36
79	Diazinon	94.42	96.59	95.97	1.94-7.37	6.96	96.6	95.54	98.29	0.52-4.31	4.1	102.61	94.05	93.37	2.72-3.74	3.5
80	Brassinolide	73.27	76.79	73.45	2.35-6.35	5.03	96.89	114.32	102.8	3.58-7.28	15.03	76.73	77.23	79.16	4.49-5.93	7.97
81	Tebuconazole	90.4	94.91	94.23	1.27-4.50	3.99	99.51	91.39	95.35	0.74-2.54	3.69	107.05	105.93	102.21	1.13-2.10	1.1
82	Pentrimazole	92.41	94.81	89.83	2.89-3.56	3.11	84.75	94.72	96.09	0.72-7.56	3.47	99.03	94.54	94.75	2.39-2.72	2.73
83	Pythiamin	92.54	95.02	90.49	1.81-4.25	3.48	97.37	96.41	92.23	1.28-2.75	4.08	102.2	92.54	94.87	1.84-2.87	1.87
84	Propiconazole	93.17	95.79	93.59	1.59-3.27	3.91	86.85	93.75	92.05	1.27-5.3	3.49	100.47	95.19	96.32	3.99-6.07	6.26
85	Pyraoxystrobin	88.35	93.55	92.26	1.34-3.58	5.28	97.21	95.86	93.63	0.9-2.51	0.82	104.67	91.77	93.28	2.37-2.81	2.83
86	Pyraclostrobin	90.83	95.59	89.53	2.61-6.72	5.55	95.62	92.41	94.25	1.59-3.54	6.83	110.29	102.75	101.61	1.63-3.02	1.62
87	Phoxim	85.98	97.94	93.41	1.06-3.63	3.16	96.9	94.56	94.35	1.86-4.52	1.86	101.58	93.26	94.78	206-2.92	2.94
88	Cyflufenamid	102.28	88.77	91.09	0.80-3.56	3.48	91.25	94.9	96.31	1.31-4.41	1.81	114.41	102.66	107.65	3.50-4.03	4.03
89	Diniconazole	91.27	89.39	94.38	2.58-2.97	4.16	93.63	90.25	94.93	2.56-4.36	3.81	101.57	92.15	93.08	2.42-4.37	3.27
90	Ametoctradin	71.37	92.83	85.23	1.19-4.17	4.32	96.76	88.5	95.72	1.93-3	0.65	92.51	82.77	85.82	1.26-2.50	2.67
91	Indoxacarb	97.72	89.39	95.12	2.14-4.66	4.57	94.27	93.66	92.72	2.81-5.73	10.55	106.74	90.64	96.55	2.99-7.25	7.1
92	Pretilachlor	91.67	94.26	95.06	0.50-1.80	7.15	93.71	99.21	92.16	0.96-1.62	0.65	100.52	94.23	94.69	1.32-2.59	1.16

93	Difenoconazole	93.29	96.6	92.2	0.91-4.15	6.9	98.31	96.79	93.84	0.72-2.82	1.39	108.01	107.59	102.41	0.62-2.85	0.67
94	Trifloxystrobin	93.7	92.82	89.95	1.09-4.42	3.6	93.06	94.82	95.44	1.57-2.8	1.74	101.93	93.22	94.63	1.51-2.57	2.02
95	Profenofos	90.21	92.81	94.23	1.51-3.90	3.51	89.15	93.17	94.55	2.36-4.49	3.16	100.27	94.91	96.56	2.36-4.37	4.26
96	Enestroburin	96.47	95.02	88.6	1.80-3.69	2.12	92.91	96.41	97.5	1.25-2.43	3.09	101.38	92.71	94.84	1.58-3.12	1.72
97	Coumoxystrobin	94.56	92.71	84.05	0.74-2.24	1.21	107.85	101.07	91.02	1.53-5.33	4.82	102.72	93.91	96.06	1.57-2.94	2.63
98	Buprofezin	87.99	89.67	84.09	1.95-2.92	1.73	91.52	98.37	91.61	1.2-2.53	3.39	100.71	94.78	95.59	1.65-4.10	2.1
99	Emamectin Benzoate	92.42	86.33	84.06	0.85-4.72	2.61	96.92	93.7	96.7	1.26-1.87	1.89	103	95.55	93.54	1.62-2.85	1.98
100	Fenpropathrin	88.78	90.93	91.37	4.82-10.17	7.4	87.08	87.16	95.56	2.19-12.9	8.14	110.1	112.46	106.46	1.64-4.02	5.42
101	Chlorpyrifos	94.59	96.89	86.67	0.70-3.59	2.15	94.77	99.23	96.11	1.24-2.96	2.55	104.08	106.67	103.44	1.91-2.40	2.58
102	Hexythiazox	94.64	94.51	91.19	1.54-3.57	2.06	93.67	93.47	94.12	2.24-2.71	1.88	98.97	95.14	95.89	2.93-3.41	4.67
103	Propargite	87.8	94.05	90.54	1.76-5.26	3.24	92.28	94.6	95.96	2.12-2.53	3.66	98.62	106.85	97.84	2.40-2.81	2.6
104	Spirodiclofen	90.34	96.21	88.15	2.14-4.83	3.93	90.23	88.95	91.58	1.61-7.83	7.4	105.83	94.84	95.86	3.31-4.35	7.69
105	Pyridaben	91.74	94.44	90	2.26-3.57	2.51	96.27	91.96	89.32	1.68-3.03	2	99.19	88	92.4	2.23-5.77	4.83
106	Carbosulfan	86.82	81.21	77.51	4.89-7.37	5.42	30.25	30.97	32.87	1.32-7.97	7.87	48.33	43.47	44.69	2.02-2.98	5.19
107	Etofenprox	97.65	97.14	97.29	1.99-4.18	5.87	97.94	87.65	95.5	1.8-3.03	3.26	95.92	89.3	95.27	3.39-4.41	4.93

Table S7. MRL of medicinal plants, *Fritillaria thunbergii* Miq, *Dendrobium officinale* Kimura et Migo, and Chrysanthemum in GB 2763-2021.

Pesticide	Medicinal plants (mg/kg)	*Fritillaria thunbergii Miq* (mg/kg)		*Dendrobium officinale Kimura et Migo* (mg/kg)		Chrysanthemum (mg/kg)	
		Fresh	Dry	Fresh	Dry	Fresh	Dry
Imidacloprid	-	0.05	0.2	2	3	1	2
Thiamethoxam	-	-	-	-	-	2	-
Difenoconazole	-	-	-	1	2	-	-
Dimethomorph	-	-	-	20	20	-	-
Acephate	0.05	-	-	-	-	-	-
Dimethoate	0.05	-	-	-	-	-	-
Carbofuran	0.02	-	-	-	-	-	-
Carbosulfan	0.02	-	-	-	-	-	-

Table S8. Residual levels (mg/kg) of tested compounds in *Fritillaria thunbergii* Miq.

**Oringins**	**Panan**	**Panan**	**Taizhou**	**Jinhua**	**Jinhua**	**Lishui**	**Panan**	**Sichuan**	**Bozhou**	**Panan**	**Panan**	**Liuan**	**Panan**	**Anhui**	**Panan**	**Jinhua**	**Sichuan**	**Jinhua**	**Anhui**	**Jinhua**
**Samples NO.** / **Pestiside**	**1**	**2**	**3**	**4**	**5**	**6**	**7**	**8**	**9**	**10**	**11**	**12**	**13**	**14**	**15**	**16**	**17**	**18**	**19**	**20**
3-Indoleacetic Acid		0.002					0.001	0.004	0.003	0.002	0.005	0.01	0.005	0.003	0.006	0.002	0.005	0.014	0.021	0.005
Propamocarb								0.001					0.007			0.002				
Gibberellic Acid		0.001						0.005	0.004	0.005	0.002	0.004	0.008	0.011	0.009	0.007	0.009	0.004	0.004	0.002
Sodium 5-Nitroguaiacolate	0.001			0.001		0.001		0.004				0.001			0.001	0.001	0.001	0.001	0.005	0.001
Sodium 2-Nitrophenolate	0.001	0.001	0.001	0.001		0.002		0.014			0.005								0.002	0.002
Sodium 4-Nitrophenolate						0.003		0.003			0.004					0.001	0.001	0.001		
2,4-D	0.015																			
Acetamiprid		0.001	0.001		0.001						0.001									
Carbendazim	0.061	0.029	0.001	0.001	0.049	0.005	0.001	0.028			0.023	0.001	0.028	0.003	0.02	0.004	0.02	0.031	0.194	0.024
Tricyclazole								0.001												
6-Benzylaminopurine													0.004	0.005	0.005	0.004	0.005	0.022	0.01	0.001
Carbofuran	0.021	0.174	0.002	0.001	0.006	0.003	0.001				0.001								0.001	0.003
Chlorantraniliprole		0.001	0.001	0.001		0.001		0.001					0.001		0.002	0.001	0.001	0.001	0.001	
Clomazone	0.003	0.001			0.001	0.004														
Azoxystrobin	0.001		0.001	0.001	0.001		0.001							0.001			0.003		0.007	0.001
Pyrimethanil								0.004								0.001	0.002	0.001	0.002	
Fenamidone								0.002						0.001			0.001	0.001	0.001	
Boscalid	0.006		0.001								0.003		0.036	0.004	0.051	0.007	0.015	0.383	0.158	0.015
Dimethomorph					0.002			0.003											0.001	
Isoprothiolane																			0.001	
Fluopicolide								0.002												
Uniconazole								0.003	0.001	0.001		0.002	0.001	0.001	0.001	0.002	0.001	0.001	0.001	0.001

Triadimefon																			0.008	
Triazophos		0.001						0.001	0.002	0.002	0.002	0.001	0.001	0.002	0.001	0.002	0.001	0.001	0.001	
Chromafenozide	0.005																			
Epoxiconazole								0.002	0.011	0.013		0.006	0.006	0.012	0.003	0.007	0.004	0.008	0.007	0.003
Picoxystrobin									0.001											
Flusilazole																	0.001			
Kresoxim-Methyl	0.005																	0.001		
Diazinon																	0.001	0.001	0.001	
Tebuconazole																0.001	0.001	0.001		
Propiconazole											0.001						0.004	0.002	0.026	0.003
Pyraclostrobin	0.004	0.003	0.004	0.004	0.004	0.003	0.003	0.003	0.003	0.003	0.004	0.003	0.003	0.011	0.003	0.003	0.01	0.032	0.084	0.009
Phoxim										0.001					0.001				0.02	
Pretilachlor	0.001				0.007															
Difenoconazole	0.001	0.001	0.001	0.001	0.002	0.001	0.001	0.002	0.001		0.001	0.001	0.001	0.002	0.001	0.001	0.013	0.015	0.083	0.004
Trifloxystrobin	0.001	0.001	0.001	0.001	0.001	0.001	0.001	0.001	0.001	0.001	0.001	0.001	0.001		0.001	0.001	0.001		0.002	0.001
Fenpropathrin	0.001			0.002		0.003		0.004		0.001	0.001		0.001		0.001		0.001		0.002	0.001
Chlorpyrifos	0.001	0.001	0.001	0.002	0.001	0.003	0.001	0.004	0.001	0.001	0.001	0.001		0.001	0.001	0.001	0.001	0.001	0.003	0.001
Bifenthrin					0.001								0.001			0.001	0.005	0.005	0.004	0.001

Table S9. Residual levels (mg/kg) of tested compounds in *Chrysanthemum Morifolium* Ramat.

Oringins	Tongxiang	Tongxiang	Tongxiang	Tongxiang	Tongxiang	Tongxiang	Tongxiang	Tongxiang	Tongxiang	Tongxiang	Tongxiang	Tongxiang	Tongxiang	Tongxiang	Tongxiang	Tongxiang	Tongxiang	Tongxiang	Tongxiang	Tongxiang
Samples NO. / Pestiside	**1**	**2**	**3**	**4**	**5**	**6**	**7**	**8**	**9**	**10**	**11**	**12**	**13**	**14**	**15**	**16**	**17**	**18**	**19**	**20**
3-Indoleacetic Acid	0.138	0.181	0.205	0.117	0.129	0.187	0.245	0.166	0.151	0.126	0.231	0.137	0.195	0.198	0.134	0.217	0.205	0.279	0.172	0.2
Dinotefuran		0.085					0.033		0.175				0.748	0.014		0.015	0.046		0.046	0.006
Propamocarb	0.006	0.009		0.009			0.024	0.042	0.006			0.007	0.061	0.008		0.032	0.01			
Methomyl		0.014			0.014	0.017	0.226	0.013					0.017				0.015			
Thiamethoxam	0.033	0.044	0.025	0.02	0.043	0.016	0.382	0.144	0.293	0.274	0.038	0.49	0.143	0.203	0.011	0.129	0.204	0.011	0.543	0.042
Gibberellic Acid	1.229	0.871	0.257	0.629	0.877	0.926	0.74	1.182	1.09	0.778	1.099	0.722	0.693	0.519	0.548	0.988	0.461	1.179	2.386	0.29
Clothianidin	0.039	0.097	0.177	0.023	0.02	0.036	0.139	0.117	0.1	0.029	0.104	0.036	0.094	0.128	0.008	0.143	0.319	0.021	0.203	0.107
Imidacloprid	0.566	0.488	0.096		1.3	0.634	0.305	0.221	0.396	0.051	0.152		0.27	0.407	0.489	0.526	1.299	0.092	0.344	0.132
Trichlorfon						0.005		0.004		0.007	0.005		0.015		0.005		0.003	0.004	0.005	
3-Hydroxycarbofuran	0.02	0.021	0.014				0.038				0.019			0.016	0.017	0.035	0.015			
Sodium 5-Nitroguaiacolate		0.003	0.001	0.012		0.006	0.004				0.003	0.009	0.006	0.001	0.006	0.006	0.004	0.032	0.024	0.005
Sodium 2-Nitrophenolate	0.007	0.005	0.009	0.016	0.013	0.014	0.008	0.007	0.009	0.021	0.011	0.015	0.008	0.009	0.017	0.009	0.012	0.239	0.031	0.011
Sodium 4-Nitrophenolate	0.009	0.007	0.008	0.017	0.012	0.012	0.007	0.007	0.001	0.019	0.008	0.012	0.006	0.006	0.016	0.008	0.012	0.225	0.032	0.009
2,4-D	0.015	0.026	0.086	0.017			0.006	0.019		0.031	0.014				0.007	0.031	0.021		0.005	0.05
Dimethoate	0.003	0.004				0.004	0.006				0.012		0.003		0.002	0.008	0.003			0.001
Acetamiprid	0.497	0.812	0.027	0.018	0.111	0.039	0.178	0.127	0.124	0.005	0.152	0.004	0.135	0.748	0.03	0.63	0.938	0.004	0.104	0.047
Carbendazim	0.67	1.67	0.034	0.075	0.182	1.646	0.257	0.399	0.311	0.051	0.02	0.013	0.337	0.56	0.717	1.004	1.059	0.015	0.011	0.041

Thiacloprid		0.004	0.007		0.005	0.006		0.067	0.003	0.005	0.009			0.003	0.003	0.004		0.003		0.007
Trinexapac-Ethyl				0.071		0.07	0.058		0.056	0.056	0.055				0.056	0.076	0.08		0.145	
Tricyclazole	0.033	0.125	0.021	0.013	0.013	0.019	0.187	0.065	0.262	0.014	0.245	0.013	2.683	0.074	0.013	0.309	0.113	0.013	0.014	0.045
Indolbutyric Acid	0.005	0.006	0.005	0.006	0.01	0.009	0.008	0.006	0.005	0.007	0.007	0.006	0.011	0.008	0.007	0.007	0.008			0.006
6-Benzylaminopurine	0.008	0.006		0.008	0.001	0.002	0.009	0.009	0.008	0.001	0.001	0.006	0.008	0.008		0.009	0.009			0.001
Carbofuran	0.029	0.016	0.012				0.051	0.011	0.011		0.021		0.025	0.016	0.02	0.096	0.017			0.01
Forchlorfenuron		0.01	0.11		0.025	0.057		0.023	0.017	0.013	0.159			0.013				0.363	0.012	0.099
Isopropyl	0.01	0.042	0.01		0.003	0.004	0.069	0.179	0.174		0.008		0.042	0.04		0.085	0.05		0.019	0.006
Chlorantraniliprole	0.024	0.021	0.018	0.001	0.002	0.002	0.004	0.047	0.034	0.002	0.009	0.002	0.006	0.011		0.184	0.065	0.003	0.133	0.04
Azoxystrobin	0.064	2.362	0.098		5.025	0.693	0.876	1.138	0.132	0.022	0.099		0.285	0.111	2.565	0.441	0.235		0.075	0.115
Methyl Jasmonate	0.095	0.157	0.22	0.006	0.417	0.415	0.086	0.25	0.112	0.057	0.948	0.005	0.044	0.069	0.019	0.045	0.043	0.009	0.607	0.181
Pyrimethanil	0.005	0.009	0.004	0.002	0.011	0.004	0.13	0.005	0.005	0.003	0.005	0.001	0.004	0.005	0.002	0.135	0.004		0.062	0.002
Boscalid																			1.295	
Dimethomorph			0.042				0.082	0.225	0.014			0.019	0.115	0.12	0.005	0.038	0.015	0.003	0.043	0.019
Mandipropamid	0.004	0.004	0.004	0.004	0.004										0.004					
Isoprothiolane	0.495	0.284	0.034	0.109	1.08	0.822	0.816	1.347	0.365	0.561	0.05	0.097	0.348	0.414	0.399	0.389	0.47		0.119	0.049
Fluopicolide	0.063	0.064	0.016	0.058	0.02	0.022	0.082	0.069	0.065	0.02	0.019	0.053	0.141	0.061	0.019	0.083	0.069	0.011		0.02
Paclobutrazol	0.001	0.015	0.003	0.001			0.002	0.003	0.002		0.009		0.015	0.002	0.001	0.003	0.002		0.057	0.005
Malathion	0.228	0.304	0.248	0.268	0.187	0.226	0.248	0.249	0.25	0.234	0.226	0.274	0.406	0.323	0.295	0.287	0.427	0.226	0.283	0.281
Triadimefon	0.017	0.027	0.006			0.027	0.011	0.049	0.024	0.025	0.024		0.063	0.063		0.165	0.388			
Triazophos					0.03			0.011					0.012	0.009		0.009	0.012			
Tetrachlorantraniliprole	0.014	0.005			0.011		0.004	0.012			0.061		0.043	0.03		0.024	0.006	0.001		
Spirotetramat																			0.071	
Tetraconazole														0.024						

Cyazofamid	0.046	0.083	0.019				0.084	0.058	0.069	0.009	0.044		0.099	0.051		0.16	0.083			0.021
Flusilazole	0.065	0.1	0.164	0.04	0.377	0.423	0.083	0.101	0.082	0.303	0.271	0.034	0.129	0.061	0.219	0.079	0.058	0.004	0.007	0.142
Kresoxim-Methyl		0.022					0.015	0.256					0.012			0.03	0.016			
Fipronil	0.067	0.065	0.057	0.003	0.022	0.024	0.072	0.183	0.086	0.007	0.091		0.191	0.068	0.003	0.092	0.117	0.003	0.003	0.052
Diazinon	0.01	0.011	0.003	0.007	0.002	0.002	0.011	0.015	0.01			0.005	0.012	0.01		0.011	0.01			0.002
Tebuconazole	0.227	0.375	0.245	0.106	0.049		0.089	0.112	0.183	0.001	0.12	1.77	0.306	0.152	0.008	0.124	0.963	0.416	0.003	0.121
Pythiamin	0.001	0.001	0.002	0.001	0.002	0.002	0.001	0.001	0.001	0.002	0.003	0.001	0.001	0.001	0.001	0.001	0.001		0.001	0.002
Propiconazole		0.035	0.014		0.029	0.049	0.08	0.175	0.042		0.095		0.26	0.018		0.051	0.017			0.012
Pyraclostrobin	0.067	0.076	0.169	0.164	0.002	0.021	0.161	0.308	0.043	0.008	0.191	1.507	0.102	0.07	0.089	0.123	0.286	0.804	0.019	0.037
Phoxim	0.049	0.252	0.085	0.003	0.016	0.009	0.18	0.115	0.058		0.073	0.002	0.042	0.048		0.172	0.041	0.012		0.073
Diniconazole	0.042	0.042		0.032	0.044	0.049	0.046	0.056	0.045	0.025	0.05	0.029	0.066	0.043	0.051	0.049	0.039		0.003	0.009
Indoxacarb	0.122	0.507	0.012	0.003	0.012	0.005	0.067	0.014	0.301	0.007	0.032	0.006	0.366	0.167	0.006	0.102	0.186		0.21	0.025
Pretilachlor	0.004	0.003	0.086	0.001			0.006	0.007	0.008			0.001	0.007	0.004		0.006	0.004			0.08
Difenoconazole	0.059	0.362	0.06	0.072	0.891	0.174	0.807	1.184	0.177	0.003	0.219	0.034	0.953	0.111	0.068	0.755	0.321	0.004	0.132	0.099
Trifloxystrobin	0.059	0.362	0.06	0.072	0.891	0.174	0.807	1.184	0.177	0.003	0.219	0.034	0.953	0.111	0.068	0.755	0.321	0.004	0.132	0.099
Profenofos	0.069	0.016	0.011		0.009	0.013	0.04	0.017	0.043		0.239		0.035	0.022		0.011	0.03			0.015
Buprofezin	0.008	0.067	0.017	0.008	0.008	0.013	0.064	0.067	0.028	0.007	0.007	0.009	0.246	0.117	0.008	0.027	0.069	0.006	0.036	0.01
Emamectin																				
Benzoate	0.02	0.025	0.003	0.002	0.004	0.004	0.009	0.021	0.006	0.002	0.004	0.105	0.026	0.018	0.002	0.012	0.025	0.004	0.003	0.005
Fenpropathrin	0.042	0.721	0.289	0.669	0.071		0.855	0.403	0.173	0.009	0.445	0.282	0.781	0.407	0.102	0.213	1.007	1.278		0.34
Chlorpyrifos	0.024	0.597	0.211	0.646	0.077	0.014	0.834	0.371	0.164	0.009	0.386	0.259	0.815	0.407	0.063	0.223	1.19	1.458	0.003	0.371
Propargite							0.006						0.010							0.005
Pyridaben	0.023	0.019		0.013			0.013	0.011	0.01		0.008	0.018	0.012	0.011		0.013	0.011			0.011
Carbosulfan							0.003		0.001		0.001		0.003		0.004	0.034	0.001			
Etofenprox		0.003			0.007		0.001				0.001			0.005		0.001				
Bifenthrin	0.006	0.026	0.005	0.002		0.006	0.019	0.03	0.048		0.007	0.031	0.005	0.071	0.002	0.03	0.026	0.002	0.825	0.009

Table S10. Residual levels (mg/kg) of tested compounds in *Dendrobium officinale* Kimura et Migo.

Oringins	Yunnan	Jinhua	Jinhua	Panan	Jinhua	Panan	Panan	Guangxi	Guangxi	Anhui	Huoshan	Jinhua	Panan	Panan	Panan	Panan	Panan	Panan	Yunnan	Yunnan
**Samples NO.** / **Pestiside**	**1**	**2**	**3**	**4**	**5**	**6**	**7**	**8**	**9**	**10**	**11**	**12**	**13**	**14**	**15**	**16**	**17**	**18**	**19**	**20**
3-Indoleacetic Acid	0.003	0.004	0.002	0.004	0.002	0.006	0.008	0.006	0.007	0.017	0.013	0.01	0.004	0.003	0.009	0.001	0.008	0.002	0.002	0.005
Propamocarb				0.486	1.95	0.004													5.715	0.003
Gibberellic Acid	0.050	0.147	0.132	0.134	0.433	0.233	0.208	0.221	0.125	0.182	0.102	0.021	0.110	0.102	0.108	0.138	0.104	0.115	0.040	0.028
Clothianidin																			0.015	0.008
Imidacloprid				0.002			0.005		0.001		0.001		0.001							
Sodium 2-Nitrophenolate						0.002		0.005		0.002	0.003									
Sodium 4-Nitrophenolate								0.004	0.002	0.002	0.004	0.002	0.002	0.002	0.002	0.001	0.001		0.003	0.002
Acetamiprid																			0.001	
Carbendazim							0.016	0.002	0.003	0.016	0.054	0.002	0.005			0.002			0.003	0.007
Thiacloprid				0.001																
Tricyclazole																			0.001	
Chlorantraniliprole	0.001	0.007	0.001		0.003		0.038			0.005	0.004	0.003	0.001	0.008		0.001	0.001	0.003	0.13	0.004
Azoxystrobin				0.005	0.001		0.047		0.001		0.012								0.143	0.004
Pyrimethanil						0.015				0.01			0.002			0.003				
Boscalid																0.002				2.09
Fluopicolide				0.061	0.175	0.002					0.001	0.001	0.001	0.001	0.001	0.001	0.001	0.001	1.005	0.005

Paclobutrazol																	0.005	
Triadimefon																	0.135	
Tetrachlorantraniliprole																	0.022	
Picoxystrobin									0.01									
Flusilazole									0.002									
Tebuconazole					1.766	0.001	0.009		0.371			0.001					0.526	0.006
Pyraclostrobin				0.001	0.438	0.001	0.001	0.002	0.144	0.002	0.002	0.002	0.006	0.002	0.004	0.002	0.002	0.585
Phoxim							0.01											
Indoxacarb			0.017															
Difenoconazole		0.006	0.014	0.002	0.007				0.006	0.001							0.001	0.003
Trifloxystrobin					0.001		0.006		0.001	0.001	0.001	0.001	0.001	0.001	0.001	0.001	0.001	0.001
Profenofos																	0.002	
Buprofezin	0.001	0.002	0.001	0.002	0.002	0.002	0.002	0.002	0.002	0.002	0.001	0.001	0.001	0.002	0.001	0.002	0.002	0.002
Fenpropathrin							0.002										0.003	
Chlorpyrifos							0.002										0.003	
Pyridaben			0.004	0.003					0.005	0.003								
